# Preserved Cerebral Oxygen Metabolism in Astrocytic Dysfunction: A Combination Study of ^15^O-Gas PET with ^14^C-Acetate Autoradiography

**DOI:** 10.3390/brainsci9050101

**Published:** 2019-05-03

**Authors:** Carla Mari Macaisa, Tadashi Watabe, Yuwei Liu, Victor Romanov, Yasukazu Kanai, Genki Horitsugi, Hiroki Kato, Eku Shimosegawa, Jun Hatazawa

**Affiliations:** 1 Department of Nuclear Medicine and Tracer Kinetics, Osaka University Graduate School of Medicine, Suita 565-0871, Japan; carlamari_md@yahoo.com (C.M.M.); liu@tracer.med.osaka-u.ac.jp (Y.L.); romanov@tracer.med.osaka-u.ac.jp (V.R.); horitsugi@tracer.med.osaka-u.ac.jp (G.H.); kato@tracer.med.osaka-u.ac.jp (H.K.); hatazawa@tracer.med.osaka-u.ac.jp (J.H.); 2 Department of Molecular Imaging in Medicine, Osaka University Graduate School of Medicine, Suita 565-0871, Japan; ykanai@mi.med.osaka-u.ac.jp (Y.K.); eku@tracer.med.osaka-u.ac.jp (E.S.)

**Keywords:** fluorocitrate, astrocyte, oxygen consumption, TCA cycle, ^15^O-gas PET, cerebral blood flow, cerebral metabolic rate of oxygen, ^14^C-acetate

## Abstract

Fluorocitrate (FC) is a specific metabolic inhibitor of the tricarboxylic acid (TCA) cycle in astrocytes. The purpose of this study was to evaluate whether inhibition of the astrocyte TCA cycle by FC would affect the oxygen metabolism in the rat brain. At 4 h after the intracranial FC injection, the rats (*n* = 9) were investigated by ^15^O-labeled gas PET to measure the cerebral blood flow (CBF), the cerebral metabolic rate of oxygen (CMRO_2_), oxygen extraction fraction (OEF), and cerebral blood volume (CBV). After the ^15^O-gas PET, the rats were given an intravenous injection of ^14^C-acetate for autoradiography. ^15^O-gas PET showed no significant differences in any of the measured parameters between the ipsilateral and contralateral striatum (high dose group: CBF (54.4 ± 8.8 and 55.3 ± 11.6 mL/100 mL/min), CMRO_2_ (7.0 ± 0.9 and 7.1 ± 1.2 mL/100 mL/min), OEF (72.0 ± 8.9 and 70.8 ± 8.2%), and CBV (4.1 ± 0.8 and 4.2 ± 0.9 mL/100 mL), respectively). In contrast, the ^14^C-acetate autoradiography revealed a significant inhibition of the astrocyte metabolism in the ipsilateral striatum. The regional cerebral oxygen consumption as well as the hemodynamic parameters were maintained even in the face of inhibition of the astrocyte TCA cycle metabolism in the rat brain.

## 1. Introduction

Astrocytes or astroglia are the largest and most numerous of the glial cells in the central nervous system; they provide support, nourishment, and protection for neurons [1]. They serve as “gatekeepers” of the neuronal energy supply [2], and also function as passive partners of neurons in the central nervous system (CNS) [3] by controlling the levels of neurotransmitters around synapses and maintaining a suitable metabolic and ionic environment for the neurons [4].

Two specific types of interactions are believed to exist between neurons and astrocytes: the glutamate–glutamine cycle and the lactate shuttle [5]. The glutamate/gamma-aminobutyric acid (GABA)/glutamine cycle is a metabolic pathway in which glutamate or GABA released from neurons is taken up by the astrocytes; in turn, astrocytes release glutamine that is taken up by the neurons for use as a precursor for the synthesis of glutamate or GABA. During ischemia, the tricarboxylic acid (TCA) cycle is suppressed in the neurons and astrocytes due to the oxygen metabolism decline [1]. In addition, the synthesis by the transamination of glutamate from α-ketoglutarate, which is one of the TCA intermediates, also ceases [1]. Therefore, the glutamine–glutamate cycle between neuronal and glial cells is necessary to maintain the glutamine–glutamate balance, especially in the ischemic brain. Glutamate uptake by glial cells normally prevents excitotoxic elevation of the glutamate levels in the brain extracellular space; this process appears to be a critical determinant of neuronal survival in the ischemic brain [1]. In the astrocyte–neuron lactate shuttle hypothesis [6], it is the astrocyte that consumes glucose through anaerobic glycolysis to synthesize pyruvate and then lactate. This lactate is secreted into the extracellular space, to be taken up by the neurons for further oxidative degradation.

Fluorocitrate (FC) inhibits aconitase, which is an enzyme of the TCA or Krebs cycle, and is taken up preferentially by astrocytes. In previous studies [1], the intrastriatal injection of FC at 1 nmol induced a significant reduction of the ^14^C-acetate uptake at 4 h after the injection of FC. Acetate was reported as a specific substrate for astrocytes via the monocarboxylate transporter-1 (MCT1) before the FC study [7]. Moreover, astrocyte dysfunction may contribute to abnormal neuronal metabolism and neurovascular coupling in disease and injury [2]. Hirose et al. also reported that FC injection resulted in an increased uptake of ^18^F-FDG (fludeoxyglucose) in the ipsilateral hemisphere, suggesting that the increased glucose utilization in the neurons was mediated by N-methyl-d-aspartate (NMDA) receptors [8]. However, oxygen consumption under the condition of astrocytic metabolic dysfunction has not been evaluated in vivo.

For the effective production of adenosine triphosphate (ATP) under aerobic conditions, oxygen is essential in the electron transfer system after the TCA cycle in the mitochondria of the brain. We have successfully established a methodology for the in-vivo measurement of cerebral oxygen metabolic parameters such as cerebral blood flow (CBF), cerebral metabolic rate of oxygen (CMRO_2_), oxygen extraction fraction (OEF), and cerebral blood volume (CBV) in rats using ^15^O-labeled gas positron emission tomography (PET) [9].

The primary purpose of this study was to evaluate whether inhibition of the astrocyte TCA cycle by FC would affect the regional oxygen metabolism in the rat brain.

## 2. Materials and Methods

### 2.1. Preparation of FC Solution

FC solution for intrastriatal injection was prepared according to the procedure described in the previous study [10]. In brief, 8.8 mg of FC Ba^2+^ salt (Sigma-Aldrich company, St. Louis, MO, USA) was dissolved in 1.1 mL of 0.1 mmol/L HCl. About three drops of 0.1 mmol/L Na_2_SO_4_ were added to precipitate the Ba^2+^. Two milliliters of 0.1 mmol/L Na_2_HPO_4_ were added; then, the suspension was centrifuged at 3000 rpm for 5 min. The supernatant (1 mL) was diluted with 2.2 mL of saline to the final concentration, and the pH was adjusted to 7.2 at the final concentration of 1 nmol/μL (high dose). For a final concentration of 0.33 nmol/μL (low dose), 100 μL of supernatant was diluted with 2900 μL of saline.

### 2.2. Animal Preparation

Male Wistar rats (*n* = 9, age = 7 to 8 weeks, body weight = 198 ± 20 g) were purchased from Japan SLC (Hamamatsu, Japan). They were housed under a 12-h light/dark cycle and provided free access to food and water. The rats were anesthetized with 2% isoflurane and placed in a stereotaxic apparatus (Narishige SR-6R-HT, Tokyo, Japan). A stainless steel needle (26-gauge with Hamilton 10 μL syringe) was inserted into the striatum, according to the atlas of Paxinos and Watson (1998); 0.2 mm anterior to the bregma, 3.2 mm lateral to the midline, and 6.0 mm below the cortical surface [11].

FC (0.33 nmol/μL in the low-dose group (*n* = 3) or 1 nmol/µL in the high-dose group (*n* = 6)) was infused through the infusion needle into the ipsilateral striatum of each rat. The infusion was performed for 4 min at a flow rate of 0.25 μL/min, and the infusion needle was left in place for an additional 3 min to reduce the reflux of infused drugs along the cannula track. At the same time, saline solution (1 μL) was infused into the contralateral striatum [11].

All the animal experiments were performed in compliance with the guidelines of the Institute of Experimental Animal Sciences. The protocol was approved by the Animal Care and Use Committee of the Graduate School of Medicine, Osaka University.

### 2.3. ^15^O-Gas PET Measurement

PET measurement using ^15^O-gas was performed according to a previously described procedure [12]. ^15^O-labeled gases were produced by an ^14^N (d, n) ^15^O reaction using the cyclotron (CYPRIS HM-12S; Sumitomo Heavy Industries Ltd., Tokyo) at an average beam current of 7 μA and a deuteron acceleration energy of 6 MeV. The flow rates and the radioactivity concentrations of the ^15^O-labeled gases were controlled by the gas concentration stabilizing system (CYPRIS G3-A; Sumitomo Heavy Industries Ltd., Tokyo). The oxygen concentration was maintained at around 30% using a gas mixture device with pure oxygen to supply the ^15^O-labeled gases [12].

Four hours after the intrastriatal FC infusion, the rats in the low-dose and high-dose groups were examined by ^15^O-labeled gas PET [9]. According to the a previously described procedure [12], the PET scanning was performed using a micro PET-computed tomography (CT) scanner (Inveon; Siemens Medical Solutions, Knoxville, USA). A polyethylene tube was inserted into the femoral artery for arterial blood sampling under 2% isoflurane anesthesia. The anesthesia was switched to intramuscular injection of xylazine (4.8 mg/kg), butorphanol (1.6 mg/kg), and midazolam (1.2 mg/kg), and a flexible plastic tube was inserted into the trachea for inhalational administration of the ^15^O-labeled gases after the tracheotomy. Artificial ventilation was started using the respirator (SN-480-7; Shinano Seisakusyo, Tokyo, Japan) (respiratory rate = 60 ventilation per min, ventilation volume = 3 mL). The rats were placed in a supine position on a bed, and their rectal temperature was automatically kept at 37.0 °C ± 0.5 °C. The heart rate, systolic blood pressure (SBP), and diastolic blood pressure (DBP) were measured using a tail-cuff type system (BP-98A-L; Softron, Tokyo, Japan) during the PET scan measurement. The PET scan was started with the inhalation start of each ^15^O-labeled gas. Using the steady-state inhalation method, (9) ^15^O-labeled gases were ventilated continuously during the 16-min PET scanning duration: the ^15^O-CO_2_ gas (200 MBq/min) for calculation of the CBF and the ^15^O-O_2_ gas (400 MBq/min) for calculation of the CMRO_2_ and OEF. In addition, ^15^O-CO gas (400 MBq/min) was also ventilated by inhalation for 3 min to calculate the CBV, and the PET scanning was continued for up to 13 min. Arterial blood samples were taken at 13 and 16 min after the start of the PET scanning in the ^15^O-CO_2_ and ^15^O-O_2_ studies, and at 10 min after the start of the PET scanning in the ^15^O-CO study. The arterial blood gas (pH, PaCO_2_, PaO_2_, SaO_2_, hemoglobin, and hematocrit) were measured using a blood gas analyzer in the ^15^O-CO_2_ and ^15^O-O_2_ PET studies. After the PET acquisition, the CT scanning was performed for scatter and attenuation correction (tube voltage of 80 kV and tube current of 140 μA). The radioactivity count and weight of the whole blood and plasma were measured using a NaI scintillation well counter [9].

### 2.4. ^14^C-Acetate Autoradiography

After the ^15^O-gas PET measurement (approximately six hours after the FC injection), the rats were administered an intravenous injection of ^14^C-acetate (1.1 MBq/rat) dissolved in saline. Five minutes later, the rats were killed by euthanasia according to a previously described procedure [1]. The brains were removed and frozen. Coronal sections (30–60 μm) were prepared in a cryostat at −20 °C and placed in contact with an imaging plate (Fuji Film Co. Ltd., Tokyo, Japan) for a week. Then, the photo-stimulated luminescence (PSL) values in each region of the autoradiograms were obtained using the Bio-Imaging Analyzer System (BAS-1500; Fuji Photo Film, Tokyo, Japan). The radioactivity concentrations in regions of interest (ROIs) were expressed using Image J application (Version 1.46) (National Institutes of Health (NIH): Bethesda, MA, USA, 2012.), and the data were expressed as the proportion of the values on the FC-injected side to that on the saline-injected contralateral side. The autoradiographic analysis was also performed in the same way in another group of rats (*n* = 3; 4 h after the time of FC injection) for validation.

### 2.5. PET Data Analysis

**Figure 1:**
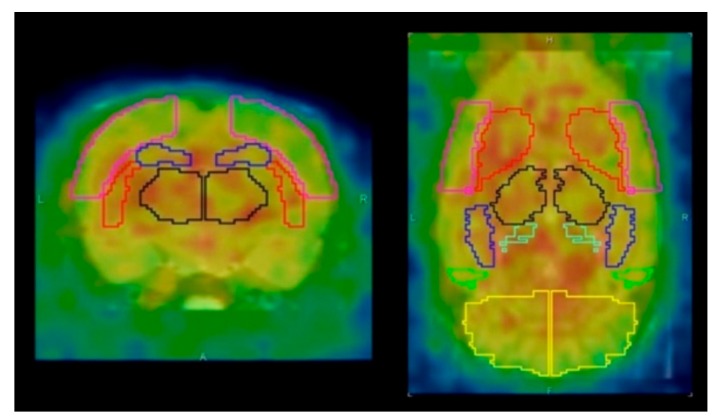
Axial and coronal positron emission tomography (PET) images with volume of interest (VOI) template (W. Schiffer). Red line area indicated the striatum. Pink line area (the cerebral cortex), black line area (the thalamus), blue line area (the hippocampus), and yellow line area (the cerebellum) were also shown.

The PET image reconstruction and data analysis were performed according to previously described methods [12]. PET images were reconstructed using ordered-subset expectation maximization 3D followed by maximum a posteriori (OSEM3D-MAP) (16 subsets, two iterations for OSEM3D, and 18 iterations for MAP) with scatter and attenuation correction. The image matrix was 128 × 128 × 159 and the voxel size was 0.776 × 0.776 × 0.796 mm. Quantitative PET images (CBF, OEF, CMRO_2_, and CBV) were generated by the steady-state ^15^O-gas inhalation method using in-house software [12]. The PET images were aligned with the template of a T2-weighted magnetic resonance (MR) image using the rigid registration method by the PMOD software, version 3.604 (PMOD Technologies, Zürich, Switzerland). The volume of interest (VOI) template (W. Schiffer) was automatically placed on the brain of the PET images (Figure 1) [12]. The quantitative values (CBF, OEF, CMRO_2_, and CBV) in the striatum were obtained for the low-dose and high-dose groups.

### 2.6. Statistics

The quantitative values were compared between the ipsilateral and contralateral sides by a paired *t*-test using Microsoft Excel 2010 (Redmond, WA, USA). *p* values of less than 0.05 were considered as denoting statistical significance.

## 3. Results

The vital signs and arterial blood gas (ABG) results during the ^15^O-gas PET are shown in Table 1. One out of the six rats in the high-dose group was excluded from the analysis due to its physically unstable condition during the ^15^O-water gas PET. In all the remaining rats, the hemodynamic parameters were all within the respective normal ranges.

**Figure 2:**
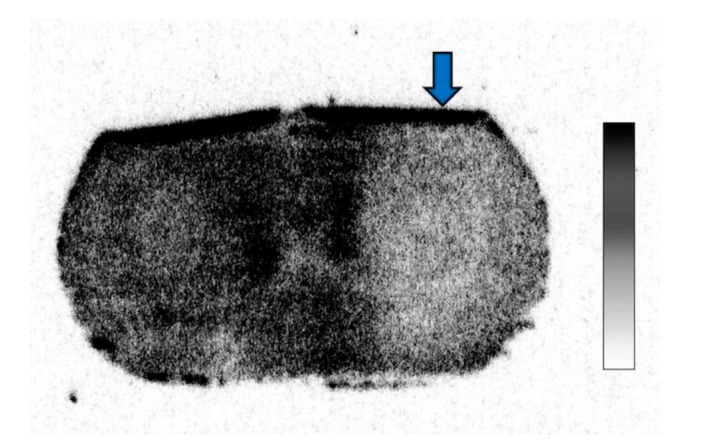
^14^C-acetate autoradiography after fluorocitrate (FC) injection showing decreased uptake in the ipsilateral hemisphere.

**Figure 3:**
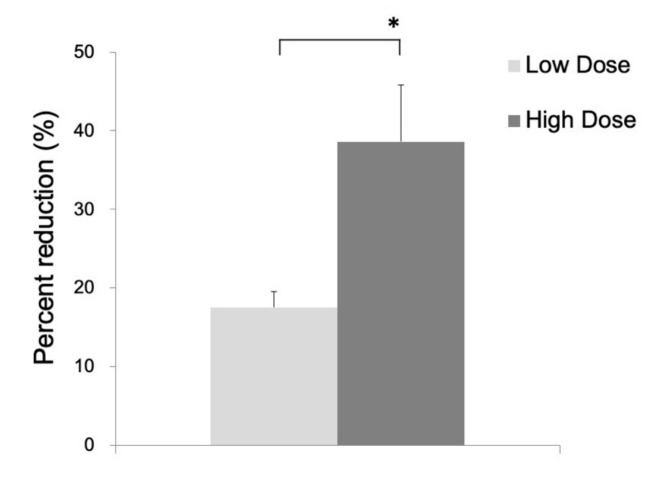
Percent reduction of ^14^C-acetate in the ipsilateral as compared to the contralateral side in the low-dose group (17.5 ± 2.0%) and high-dose group (38.6 ± 7.2%). Data are expressed as mean ± standard deviation. (*, *p* < 0.05).

In the ^14^C-acetate autoradiography, the uptake was reduced in the ipsilateral striatum, indicating significant inhibition of the astrocyte metabolism after FC injection (Figure 2). No reduced uptake was noted in the contralateral striatum injected with saline. As shown in Figure 3, the percent reduction of uptake was 17.5 ± 2.0% in the low-dose group (*n* = 3) and 38.6 ± 7.2% in the high-dose group (*n* = 6).

**Figure 4:**
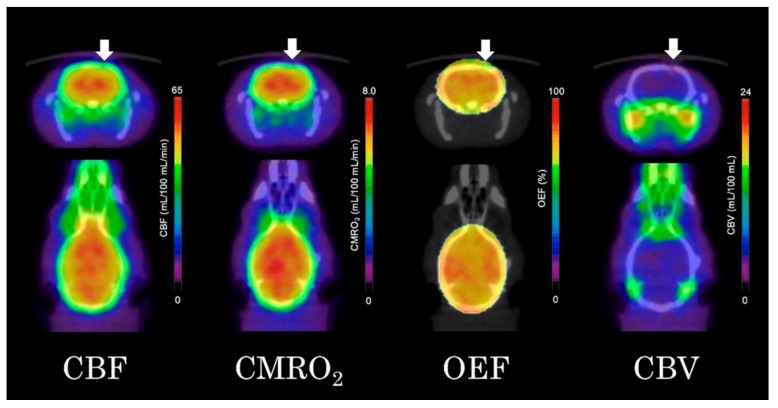
Quantitative ^15^O-gas positron emission tomography (PET) images of the cerebral blood flow (CBF), cerebral metabolic rate of oxygen (CMRO_2_), oxygen extraction fraction (OEF) and cerebral blood volume (CBV) (arrows indicated the ipsilateral side of the fluorocitrate (FC) injection).

**Figure 5:**
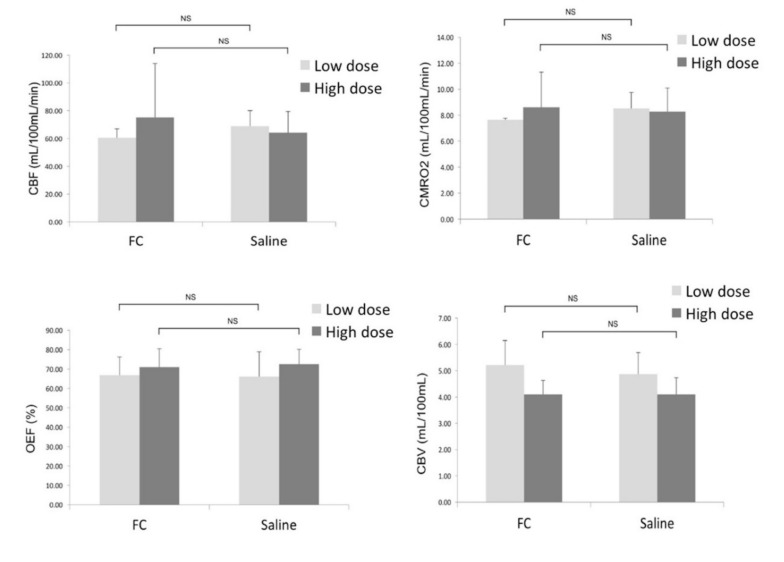
Comparison of the CBF, CMRO_2_, OEF, and CBV between the ipsilateral side (FC injection) and contralateral (saline injection) sides. Data are expressed as mean ± standard deviation. (NS: not significant).

As shown in Figure 4, the quantitative parametric PET images (CBF, OEF, CMRO_2_, and CBV) showed no significant differences between the ipsilateral striatum and contralateral striatum. VOI analysis of ^15^O-gas PET images also showed no significant differences in any of the measured parameters between the ipsilateral and contralateral striatum (Figure 5). The results in the high-dose group were as follows: (ipsilateral and contralateral striatum: CBF (54.4 ± 8.8 and 55.3 ± 11.6 mL/100 mL/min), CMRO_2_ (7.0 ± 0.9 and 7.1 ± 1.2 mL/100 mL/min), OEF (72.0 ± 8.9 and 70.8 ± 8.2%), and CBV (4.1 ± 0.8 and 4.2 ± 0.9 mL/100 mL), respectively).

## 4. Discussion

In the present study, we found that astrocyte dysfunction induced by the intrastriatal injection of FC had no significant effect on the regional cerebral blood flow or oxygen metabolism as measured by ^15^O-gas PET and ^14^C-acetate autoradiography. Parametric images of CBF, OEF, CMRO_2_, and CBV revealed no significant differences, either visually or numerically, between the ipsilateral and contralateral striatum of the rat brain, indicating preserved oxygen consumption of the neurons even in the face of astrocyte dysfunction.

Astrocytes control various neuronal functions. They have critical roles in ion buffering and in metabolic and trophic support to neurons [13]. Furthermore, they actively participate in synaptic transmission by releasing neurotransmitters such as glutamate and ATP [10]. It has been shown that the activity of glutamatergic neurons increases the concentration of extracellular glutamate that is taken up by astrocytes via sodium-dependent glutamate transporters, resulting in an increase in the concentration of Na^+^ in the astrocytes. This in turn results in stimulation of the Na^+^/K^+^-ATPase activity [14].

In our study, the microinfusion of FC, a specific inhibitor of glial metabolism, caused a significant reduction of the ^14^C-acetate uptake in the infused and surrounding regions. We injected FC into the striatum according to the previous study by Hosoi et al. [11]. In their study [11], a greater than 80% decrease of the ^14^C-acetate uptake in the striatum was observed at 4 h after intrastriatal injection of 1 nmol/L of FC. While our study did not show such an intense decrease as in previous studies, a significant reduction was observed in the ipsilateral striatum. The decrease was high enough to cause astrocyte dysfunction, which lead to significant changes in the cerebral metabolism. Furthermore, in this study, even at 6 h after the FC injection, the inhibition was still observed, regardless of the dose administered. Acetate is preferentially taken up into astrocytes by MCT-1-mediated transport [15]. ^14^C-acetate is converted to ^14^C acetyl CoA, and then incorporated into the TCA cycle as a substrate in the mitochondria. Therefore, the marked reduction of ^14^C-acetate uptake in this study was considered as being due to transient mitochondrial dysfunction [15].

Our group reported the normal values of ^15^O-gas PET using normal rats (CBF: 62.1 ± 19.6 mL/100 mL/min, CMRO_2_: 8.30 ± 0.66 mL/100 mL/min, OEF: 67.5 ± 8.1%, CBV: 4.17 ± 0.29 mL/100 mL) [12]. CBF, CMRO_2_, and OEF values in both low-dose and high-dose groups were within the range of normal values, and CBV in the low-dose group showed a larger trend compared to the normal value. However, there is no significant statistical difference in CBV between low-dose and high-dose groups. A recent study reported that a single severe traumatic brain injury induced a decreased fractional anisotropy on magnetic resonance imaging and histopathological white matter loss in the contralateral side [16]. There is a possibility that FC injection in the ipsilateral side will affect the cerebral circulation and metabolism in the contralateral side. The global effect after unilateral FC injection should be further investigated in the future study.

Neurometabolism is postulated to be a strictly oxidative (i.e., oxygen-depending) process. The basis is the higher efficiency of ATP production from glucose oxidation in the mitochondria (oxidative process) as compared to that from glycolysis (non-oxidative process; i.e., lactate-producing) [5]. Consistent with their higher energy requirements, neurons sustain a high rate of oxidative metabolism as compared to glial cells [5]. Astrocytes also convert synaptically reclaimed glutamate to glutamine, which is returned to the neurons for glutamate salvage or oxidation [2].

During physiological activation, postsynaptic glutamate uptake in the astrocytes is coupled with sodium influx [17]. The energy required for the Na1/K1 adenosine triphosphatase pumps to restore ion homeostasis is provided by glycolysis in the astrocytes, resulting in the production of lactate, which is consumed aerobically by the neurons in the Krebs cycle. The cerebral metabolic rate of glucose measured by PET during activation reflects increased astrocytic glycolysis, whereas the CMRO_2_ represents O_2_ consumption in the neurons [18].

**Figure 6:**
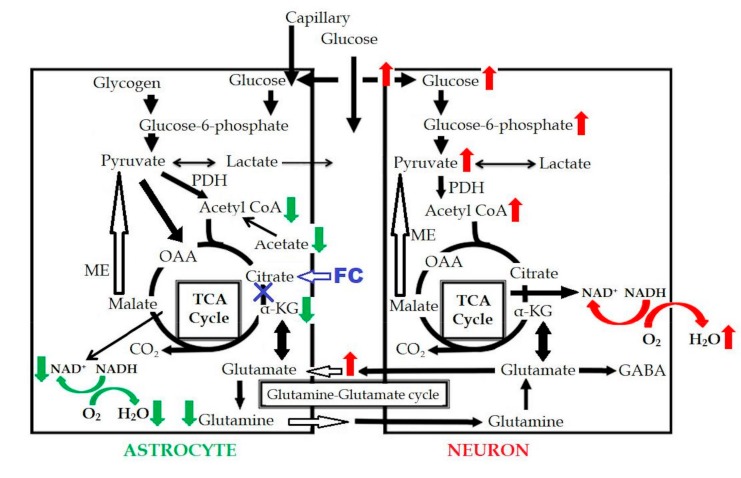
Schema of FC-induced change in the astrocytic metabolism. Red arrows indicated the increased metabolism, green arrows indicated the decreased metabolism, and blue “X” indicated the metabolic inhibition. OAA: oxaloacetate; GABA: gamma-aminobutyric acid; PDH: pyruvate dehydrogenase.

In a previous study [19], oxygen consumption in the astrocytes was found to be reduced following inhibition of the astrocyte TCA cycle. FC injection induced an increase of the extracellular glutamate level due to the decreased uptake by the astrocytes, which induced an excitotoxic effect on the neurons, in turn, resulting in increased glucose uptake and increased oxygen consumption in the neurons. In our study, no significant difference was observed in the CMRO_2_ on PET. Based on these results, the reduced oxygen consumption in the astrocytes is considered as being almost equal to the increased oxygen consumption in the neurons, as can be seen in Figure 6. This could be one of the reasons why the CMRO_2_ level was maintained in our study, even in the presence of astrocyte dysfunction.

One of the rats, which was excluded from the study, showed convulsive seizures after the injection of FC (percent reduction of ^14^C-acetate uptake was 31.5%). This result may indicate that focal astrocyte dysfunction could lead to focal epileptiform discharges and sometimes to convulsive seizures, and that the process possibly depends on effects mediated by gap junctions [20]. It also suggests that the extracellular increase of glutamate triggered the convulsive seizures in the rats after the intrastriatal injection of FC.

Regarding the variability of CBF and CMRO_2_ in the FC injected side, the VOI analysis was robust enough to show the accurate results as we used a rigid registration method with an automatic VOI template. The CBF and CMRO_2_ in the contralateral side (saline-injected side) were comparable to the values of our previous study [12]. Higher variation in the FC side might be the variation in physiological response to FC as the methodology of ^15^O-gas PET is the in-vivo quantification.

This study had several limitations. Firstly, we did not measure the lactate or extracellular glutamate levels in the brain after FC injection. MR spectroscopy or microdialysis analysis should be performed in the next study to clarify the mechanism of preserved oxygen metabolism in the presence of astrocyte dysfunction. Secondly, evaluation of the symptomatic features such as convulsive seizures, was insufficient in this study; their relationship to astrocyte dysfunction and oxygen metabolism should be analyzed in future studies.

## 5. Conclusions

Regional cerebral oxygen consumption as well as hemodynamic parameters were maintained in the animal model, even in the face of inhibition of the astrocyte TCA cycle in the rat brain. Further studies are needed to clarify the detailed mechanisms of release of the transmitters in the presence of astrocyte dysfunction.

## Figures and Tables

**Table 1:** Arterial blood gas data and vital signs during the PET measurement. DBP: diastolic blood pressure, SBP: systolic blood pressure.

Parameters	Average ± Standard Deviation
pH	7.44 ± 0.05
pCO2 (mmHg)	40.4 ± 6
pO2 (mmHg)	111.2 ± 28
sO2 (%)	98 ± 1
Hematocrit (%PCV)	41.1 ± 2.0
Hemoglobin (g/dL)	14.0 ± 0.9
Heart rate (bpm)	360 ± 51
SBP (mmHg)	131 ± 29
DBP (mmHg)	102 ± 20
